# Glyco-centric lectin magnetic bead array (LeMBA) − proteomics dataset of human serum samples from healthy, Barrett׳s esophagus and esophageal adenocarcinoma individuals

**DOI:** 10.1016/j.dib.2016.03.081

**Published:** 2016-04-01

**Authors:** Alok K. Shah, Kim-Anh Lê Cao, Eunju Choi, David Chen, Benoît Gautier, Derek Nancarrow, David C. Whiteman, Peter R. Baker, Karl R. Clauser, Robert J. Chalkley, Nicholas A. Saunders, Andrew P. Barbour, Virendra Joshi, Michelle M. Hill

**Affiliations:** a The University of Queensland Diamantina Institute, The University of Queensland, Translational Research Institute, Brisbane, Queensland, Australia; b School of Veterinary Science, The University of Queensland, Gatton, Queensland, Australia; c School of Information and Communication Technology, Griffith University, Brisbane, Queensland, Australia; d QIMR Berghofer Medical Research Institute, Brisbane, Queensland, Australia; e Mass Spectrometry Facility, Department of Pharmaceutical Chemistry, University of California, San Francisco, CA, USA; f Proteomics Platform, Broad Institute of MIT and Harvard, Cambridge, MA, USA; g School of Medicine, The University of Queensland, Brisbane, Queensland, Australia; h Ochsner Health System, Gastroenterology, New Orleans, LA, USA

**Keywords:** Proteomics, Glycoprotein, Biomarker, Esophageal adenocarcinoma, Barrett׳s esophagus

## Abstract

This data article describes serum glycoprotein biomarker discovery and qualification datasets generated using lectin magnetic bead array (LeMBA) – mass spectrometry techniques, “Serum glycoprotein biomarker discovery and qualification pipeline reveals novel diagnostic biomarker candidates for esophageal adenocarcinoma” [1]. Serum samples collected from healthy, metaplastic Barrett׳s esophagus (BE) and esophageal adenocarcinoma (EAC) individuals were profiled for glycoprotein subsets via differential lectin binding. The biomarker discovery proteomics dataset consisting of 20 individual lectin pull-downs for 29 serum samples with a spiked-in internal standard chicken ovalbumin protein has been deposited in the PRIDE partner repository of the ProteomeXchange Consortium with the data set identifier PRIDE: PXD002442. Annotated MS/MS spectra for the peptide identifications can be viewed using MS-Viewer (〈http://prospector2.ucsf.edu/prospector/cgi-bin/msform.cgi?form=msviewer〉) using search key “jn7qafftux”. The qualification dataset contained 6-lectin pulldown-coupled multiple reaction monitoring-mass spectrometry (MRM-MS) data for 41 protein candidates, from 60 serum samples. This dataset is available as a supplemental files with the original publication [1].

**Specifications table**

**Table:** 

Subject area	Biology
More specific subject area	Glyco-centric proteomics analysis for serum biomarker discovery and qualification
Type of data	Table, Figure, Graph, Western-blot images
How data was acquired	The data for the biomarker discovery screen was acquired using an Agilent 6520 quadrupole time of flight (QTOF) coupled with a Chip Cube and 1200 HPLC. The targeted proteomics for the biomarker qualification was performed on an Agilent Technologies 6490 triple quadrupole mass spectrometer coupled with a 1290 standard-flow infinity UHPLC fitted with an electrospray ionization source.
Data format	Raw, processed and analyzed.
Experimental factors	Denatured serum samples (50 μg of protein per lectin pulldown) were spiked with an internal standard chicken ovalbumin (10 pmol per lectin pulldown), reduced and then alkylated [1].
Experimental features	Using semi-automated high-throughput workflow lectin magnetic bead array (LeMBA) [1], [2], [3], glycoproteins were enriched from serum samples using lectin coated magnetic beads (20 individual lectin-beads for biomarker discovery and 6 individual lectin-beads for biomarker qualification). The lectin pull-downs were subjected to on-bead trypsin digestion followed by mass spectrometric analyses for protein identification and relative quantitation.
Data source location	UQ Diamantina Institute, Translational Research Institute, Brisbane, Queensland, Australia.
Data accessibility	Data available within this article. The proteomics data can be accessed through the ProteomeXchange Consortium via the PRIDE partner repository with the data set identifier PRIDE: PXD002442.

**Value of the data**

• Serum glycoprotein sub-fraction according to lectin binding to 20 different lectins, for 3 patient groups from healthy, Barrett׳s esophagus and esophageal adenocarcinoma.
• Label free quantitation in relation to an internal standard protein across 1054 mass spectrometric runs.
• The data can be used to compare lectin-pulldown proteomes from different serum samples/conditions.

## 1 Data

Raw QTOF spectra, searched peptide-spectrum matches and protein level quantitation for serum proteins isolated by binding to each of 20 lectins per serum sample for biomarker discovery. Peptide and protein level quantitation for serum proteins isolated by 6 individual lectin per serum sample for biomarker qualification. The serum samples have been categorized to healthy, Barrett׳s esophagus or esophageal adenocarcinoma according to clinical information.

## 2 Experimental design, material and methods

**Fig. 1:**
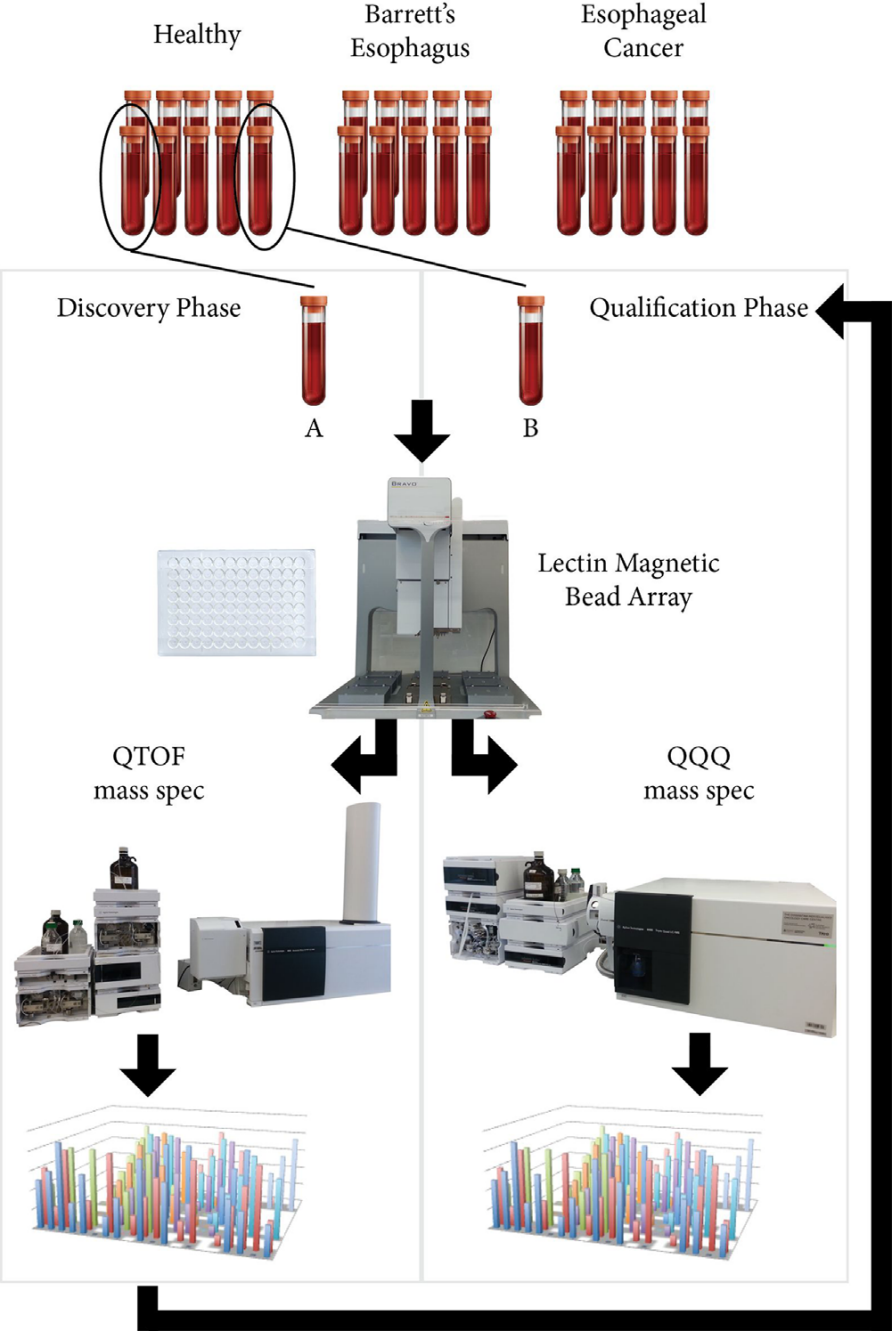
Workflow for data acquisition. Individual serum samples from patient cohorts were subjected to lectin magnetic bead array pulldown before mass spectrometry analysis. Discovery data were obtained using 20 different lectins, and analyzed by QTOF mass spectrometer with an internal reference protein between samples. Qualification data were obtained using 6 different lectins and analyzed by QQQ mass spectrometer using a scheduled MRM assay [1].

To profile differentially glycosylated serum proteins between disease conditions, each serum sample was subjected to parallel pulldown using 20 different lectins, prior to on-bead tryptic digest and LC-MS analysis (Fig. 1). The lectins used are: AAL, BPL, ConA, DSA, ECA, EPHA, GNL, HAA, HPA, JAC, LPHA, MAA, NPL, PSA, SBA, SNA, STL, UEA, WFA and WGA [2].

### 2.1 Serum sample collection

The study was approved by The University of Queensland Human Ethics Committees. Serum samples from healthy, Barrett׳s esophagus (BE) and esophageal adenocarcinoma (EAC) individuals were collected as a part of ACS [4] and SDH [5] research programs, with written informed consent. Serum from 10 ml of whole blood was processed and stored at −80 °C until use. Typically, samples were thawed once for protein estimation and simultaneously denatured. The serum samples used for the biomarker discovery phase (Healthy-9, BE-10 and EAC-10) and the biomarker qualification study (Healthy-20, BE-20, EAC-20 and population control-19) were age and gender matched.

### 2.2 Sample preparation and LeMBA pull-down

Serum samples were denatured, spiked with 10 pmol chicken ovalbumin per lectin pull-down as an internal standard, reduced, and alkylated prior to Lectin magnetic bead array (LeMBA). LeMBA and on-bead tryptic digestion was performed as describe previously using a Bravo liquid handler [1], [2], [3]. LeMBA – MS/MS was performed for biomarker discovery while LeMBA – MRM-MS was performed for the biomarker qualification stages.

### 2.3 Mass spectrometric analyzes and data processing

For biomarker discovery, samples were subjected to data dependent mass spectrometric analyzes using nano-flow LC-MS/MS (1200 HPLC, Agilent Technologies) coupled with an Agilent 6520 quadrupole time of flight [QTOF] with a Chip Cube interface. Out of total 20 µl of trypsin digested sample in 0.1% v/v formic acid, varying amount according to individual lectin pull-down was injected for mass spectrometric analyzes. Those were 9 μl for HAA, HPA and UEA, 6 μl for NPL, STL, GNL, 5 μl for BPL, DSA, ECA, MAA, SBA, WFA, and WGA, 4 μl for AAL, SNA, LPHA, PSA and JAC, 1 μl for EPHA and ConA. In total, 609 samples [(20 lectins+empty beads)×29 samples)] were processed across 8×96 well-plates and run on the mass spectrometer taking up approximately 1000 h of the instrument time. The data were extracted and searched against the Swiss-prot human database containing 20,242 entries (release 3rd Jan 2012) using the Spectrum Mill MS proteomics workbench (Agilent Technologies, Rev.B.04.00.127). Raw data (.d files), processed files (pepXML and.pkl files), and analyzed data (.xlsx) can be accessed through the ProteomeXchange Consortium [6] via the PRIDE [7] partner repository with the data set identifier PRIDE: PXD002442. The annotated spectra have been made available through the MS-Viewer (〈http://prospector2.ucsf.edu/prospector/cgi-bin/msform.cgi?form=msviewer〉) [8] and can be accessed using search key “jn7qafftux”. The data made available through PRIDE and MS-Viewer are named using the format “yyyymmdd_initials_lectin abbreviation-sample number”. In addition, the data can be accessed through GlycoSelector (〈http://glycoselector.di.uq.edu.au/index.php〉) where readers can process and visualize these data using tools available within GlycoSelector. The patient information provided in Supplementary Table 1 can be used for data processing, particularly to categorize the raw data into patient groups.

For biomarker qualification, an MRM-MS assay was set up on an Agilent Technologies 6490 triple quadrupole mass spectrometer coupled with a 1290 standard-flow infinity UHPLC and fitted with a standard-flow ESI (Jet Stream). The assay quantified 41 protein candidates incorporating a total of 140 peptides (2–5 peptides per protein) and 426 transitions (≥2 transitions per peptide) (Supplemental Table 6 of Shah et al. [1]). A 34 min long chromatographic method (24 min of actual gradient) was enough to accommodate all the transitions. The data visualization and peak integration steps were performed using Skyline version 2.1.0.4936 [9]. Six (AAL, EPHA, JAC, NPL, PSA, and WGA) out of 20 lectins were chosen for LeMBA pull-down. 79 samples including healthy, BE, EAC with additional population controls were processed using LeMBA-MRM-MS (6 lectins×79 samples=474 samples). The peptide level data were also converted into protein intensities. Proteins for which more than 50% of the peptides did not show a Pearson correlation coefficient of more than 0.6 were removed from the data set. For protein quantification, peptide(s) that did not show a Pearson correlation coefficient >0.6 with the majority (>50%) of the measured peptides from the same protein were eliminated as outliers. Equal weight was given to each peptide irrespective of its absolute intensity when calculating a normalized protein intensity. A total of 238 lectin-protein candidates were quantified. The normalized peptide-level intensity data are given in an Excel file as Supplemental Table 7 of Shah et al. [1]. Supplementary Table 2 incorporates details of samples used for biomarker qualification.

The datasets were normalized according to internal standard chicken ovalbumin responses. For biomarker discovery, at least three ovalbumin peptide intensities were selected to calculate the normalized response. For biomarker qualification, a two-step normalization approach was undertaken. In first step, the datasets were adjusted for mass spectrometric variations using isotopically labeled ovalbumin peptide. While second step normalization using internal standard chicken ovalbumin peptide accounted for variations in sample handling and lectin pull-downs. Collectively the data generated using LeMBA-LC-MS/MS, and LeMBA-LC-MRM-MS are available either via public repositories or along with the original publication [1].

## Footnotes

Appendix A Supplementary data associated with this article can be found in the online version at doi:10.1016/j.dib.2016.03.081.
